# High-throughput methods to identify male *Cannabis sativa* using various genotyping methods

**DOI:** 10.1186/s42238-022-00164-7

**Published:** 2022-11-02

**Authors:** Anthony Torres, Christopher Pauli, Robert Givens, Jason Argyris, Keith Allen, Amparo Monfort, Reginald J. Gaudino

**Affiliations:** 1 Front Range Biosciences, Lafayette, USA; 2 Centre for Agriculture and Genomics Research, Barcelona, Spain

**Keywords:** Sex marker, High-resolution melt (HRM), Genotyping, Loop-mediated isothermal amplification (LAMP), Male-associated DNA marker in *Cannabis* 2 (MADC2), DNA, Recombinase polymerase amplification (RPA), Male *Cannabis* plant

## Abstract

**Background:**

*Cannabis sativa* is a primarily dioecious angiosperm that exhibits sexual developmental plasticity. Developmental genes for staminate male flowers have yet to be elucidated; however, there are regions of male-associated DNA from *Cannabis* (MADC) that correlate with the formation of pollen producing staminate flowers. MADC2 is an example of a PCR-based genetic marker that has been shown to produce a 390-bp amplicon that correlates with the expression of male phenotypes. We demonstrate applications of a cost-effective high-throughput male genotyping assay and other genotyping applications of male identification in *Cannabis sativa*.

**Methods:**

In this study, we assessed data from 8200 leaf samples analyzed for real-time quantitative polymerase chain reaction (qPCR) detection of MADC2 in a commercial testing application offered through Steep Hill Laboratories. Through validation, collaborative research projects, and follow-up retest analysis, we observed a > 98.5% accuracy of detection of MADC2 by qPCR. We also carried out assay development for high-resolution melting analysis (HRM), loop-mediated isothermal amplification (LAMP), and TwistDx recombinase amplification (RPA) assays using MADC2 for male identification.

**Results:**

We demonstrate a robust high-throughput duplex TaqMan qPCR assay for identification of male-specific genomic signatures using a novel MADC2 qPCR probe. The qPCR cycle quotient (Cq) value representative of MADC2 detection in 3156 males and the detection of tissue control cannabinoid synthesis for 8200 samples and the absence of MADC2 detection in 5047 non-males demonstrate a robust high-throughput real-time genotyping assay for *Cannabis*. Furthermore, we also demonstrated the viability of using nearby regions to MADC2 with novel primers as alternative assays. Finally, we also show proof of concept of several additional commercially viable sex determination methodologies for *Cannabis sativa*.

**Discussion:**

In industrial applications, males are desirable for their more rapid growth and higher quality fiber quality, as well as their ability to pollinate female plants and produce grain. In medicinal applications, female cultivars are more desirable for their ability to produce large amounts of secondary metabolites, specifically the cannabinoids, terpenes, and flavonoids that have various medicinal and recreational properties. In previous studies, traditional PCR and non-high-throughput methods have been reported for the detection of male cannabis, and in our study, we present multiple methodologies that can be carried out in high-throughput commercial cannabis testing.

**Conclusion:**

With these markers developed for high-throughput testing assays, the *Cannabis* industry will be able to easily screen and select for the desired sex of a given cultivar depending on the application.

**Supplementary Information:**

The online version contains supplementary material available at 10.1186/s42238-022-00164-7.

## Background

*Cannabis sativa* is an annual plant in the Cannabaceae family that contains both monoecious and dioecious cultivars; however, in most commercial industrial and medicinal applications, dioecious cultivars are generally preferred. The species can be divided into two types, fiber type (or hemp) used for its fiber or seed oils and drug type (marijuana) that are used for secondary metabolite production (Small and Cronquist 1976; McPartland and Guy 2017; van Velzen and Schranz 2021). For medicinal or drug-type applications, female (sinsemilla) plants are desired for their increased cannabinoid production compared to past monoecious varieties that would produce self-pollinated seed that detracts from medically valued cannabinoid production (Abrams 2017). In industrial and breeding applications, male plants are desired for their more rapid growth, higher fiber quality, and ability to pollinate female plants to produce seed or a grain crop (Salentijn et al. 2019; Flajšman et al. 2021).

With primarily dioecious cultivars being grown, the segregation of males, or pollen bearing, staminate flower containing plants from female plants is essential for large-scale producers and breeders. Thus, the need for a high-throughput screening methodology to rapidly identify these males after seed germination has been amplified over the past years with the legalization of hemp in the farm bill 2019 (Graddy-Lovelace et al. 2020) and simultaneous state-led legalization of recreational *Cannabis* (Yu et al. 2020; Mandolino et al. 1999; Dills et al. 2021). *Cannabis* carries an XY/XX sex determination system (McKernan et al. 2020; Prentout et al. 2020a; Prentout et al. 2021; Prentout et al. 2020b; Mandolino et al. 2002; Vergara et al. 2016); however, it has also been proposed that it may also use an X-to-autosome balance system like other flowering plants (Ming et al. 2011; Ming et al. 2007; Punja and Holmes 2020; Razumova et al. 2016). With the presence of hermaphroditic and monoecious populations that carry an XX genotype, further research is needed to elucidate the main genetic factors contributing to sex determination, although the presence of a Y chromosome found in our work supports previous work suggesting it as a reliable genetic marker to identify phenotypic males (Mandolino et al. 1999; McKernan et al. 2020; Prentout et al. 2020a; Prentout et al. 2021; Prentout et al. 2020b; Mandolino et al. 2002; Punja and Holmes 2020; Razumova et al. 2016; Faux et al. 2014; Divashuk et al. 2014).

Previous research has focused on developing male-associated PCR markers (Mandolino et al. 1999; Mandolino et al. 2002; Techen et al. 2010)); however, during this time, the lack of an assembled Y chromosome and limited diversity of dioecious germplasm readily available limited the application of this work in commercial applications. Our research has validated and expanded this work through testing a diverse set of medicinal and recreational varieties from the California, Colorado, and European markets. This work has led us to understand there are two distinct genotypes of this male-associated DNA from *Cannabis* (MADC) 2 region, which correlated with sexual dimorphism and have been termed “male positives” and “non-male negatives.”

Using a PacBio long read sequenced male along with targeted sequencing of 48 non-related male genotypes, a male-specific genomic signature was revealed that matches the canonical MADC2 (Mandolino et al. 1999; Mandolino et al. 2002). This observation prompted us to investigate the role of multiple methodologies for identifying male *Cannabis* and explored alternative processes such as HRM, LAMP, and TwistDx genetic testing platforms to explore variants in MADC2 homology and their ability to distinguish male genotypes from non-males. All platforms are developed and tested using this locus, and nearby loci were able to successfully distinguish the two classes of the male-specific PCR amplicon from each other and from products templated by DNA of plants that bore pistillate flowers, thus providing a convenient, economical, and robust saturation-point assay for male DNA in *Cannabis* seedlings.

## Materials and methods

### Plant material

The *Cannabis* samples used in our validation work were obtained through anonymous samples submitted to Steep Hill Laboratories for genetic sex testing as part of the GenKit offering. We established an initial set of 20 samples for assay development and validation. The plants were propagated from seed grown, and phenotypic observations for sex were collected as previously described (Faux et al. 2014). Subsequently, 8200 leaf samples were used for this analysis which includes the main 3 types of *Cannabis* (types 1, 2, and 3) from various related and unrelated lineages (Gray et al. 2016; Fetterman et al. 1971; Clarke and Merlin 2013; Small and Beckstead 1973a; Small and Beckstead 1973b). These samples, anonymously submitted to Steep Hill Laboratories and presumably of California/USA origin, were assigned with unique identifiers, thereby masking any cultivar/customer-related information. Additionally, our collaborators performed a second validation experiment of our HRM and qPCR assays on a set of European *Cannabis* leaf samples from plants that were cultivated at the Centre of Genomics and Agricultural Research (CRAG) in Barcelona. The plants were phenotyped for sex as previously described (Razumova et al. 2016). The European cultivars were obtained from a known diverse lineage set, containing 158 cultivars of various drug-type hemp, fiber-type hemp, and grain-type hemp. We calculated an estimated percent accuracy of MADC2 detection of > 98.5%. The value was calculated from the retest rate and result from the 8200 sample test set (data not shown) and confirmed through phenotypic characterization of plants through assay validation (Supplemental Table 4) and collaborative research projects (Supplemental Table 2). The Y chromosome containing genome assemblies used within our analyses included Jamaican lion father (JL_Father) (BioProject no.: PRJNA575581, Medicinal Genomics, Beverly, MA) and Pineapple Banana Bubba Kush (PBBK) (BioProject no.: PRJNA378470, Steep Hill, Berkeley, CA, USA).

### DNA isolation

Genomic DNA was isolated from *Cannabis* samples using the Qiagen DNA Easy Plant genomic DNA isolation kits using manufacturer’s instructions (Qiagen, Redwood City, CA, USA) and Promega Wizard genomic DNA kit (Promega, Madison, WI, USA) using manufacturer’s instructions, as well as a more cost-effective method using FTA PlantSaver cards (GE Healthcare, Chicago, IL, USA) that used a preparation of crude genomic DNA. Crude DNA extract was prepared by Tris/Triton-X pre-treatment of 1-mm raw leaf or leaf-imprinted FTA card sections, as modified from Klimyuk et al., in a modified 96-well format for high-throughput processing (Klimyuk et al. 1993). Leaf or FTA selections were placed aseptically in a 96-well microtiter plate, 100 μL 0.25M Tris-HCl with 0.25% Triton-X-100 was added to each well, and the plates were incubated at 100 °C for 5 min, on the Veriti Thermal Cycler(ABI Biosystems, Waltham, MA, USA). A total of 3 μL of crude genomic DNA extract was used as input for pre-amplification PCR reaction. DNA isolated from the kit-based extractions did not require a pre-amplification step due to the higher quality and purity of DNA obtained.

### Pre-amplification PCR

A 10-cycle pre-amplification PCR step was introduced to the protocol to reduce the effect of plant materials in subsequent reactions, including plant pigments and potentially real-time qPCR inhibiting compounds often found in leaf extracts. A total of 2.5 μL of crude genomic DNA extract was transferred to a second PCR plate with each well preloaded with 22.5 μL of pre-amplification PCR master mix prepared per reaction as follows for either qPCR or HRM assays: 12.5 μL 2× Promega Colorless GoTaq (Promega, Madison, WI, USA), 3 μL of 4 μM qPCR mix of (MADC2 Fwd + MADC2 Rev and TC Fwd + TC Rev) or one of the following HRM mixes (Choco Mando Fwd + Choco Mando Rev, Break 1 Fwd + Plus 9 Rev, MADC2 Fwd + MADC2 Rev, or –403 Fwd + –237 Rev) (Table 1), and 7 μL nuclease-free water (Ambion, Austin, TX, USA). The reactions were subjected to the following thermocycler protocol: 1 cycle of 95 °C for 10 min, 10 cycles of 95 °C for 40 s, 60 °C for 2 min, 72 °C for 2 min, 1 cycle of 72 °C for 5 min, and then an indefinite 4 °C hold.

**Table 1:** Table of primers: primers used in the pre-amplification, LAMP, RPA, qPCR, and HRM reactions. Primer’s sequences for male detection. The following primers are from 9 and 15: MADC2 Fwd and MADC2 Rev. The other primers listed are novel designs that target the same amplicon region or a nearby loci to gain the same discrimination between males and non-males. The first column designates the type of end-point assay to be used that include loop-mediated isothermal amplification (LAMP), recombinase polymerase amplification (RPA), quantitative polymerase chain reaction (qPCR), and high-resolution melting (HRM). All sequences are provided in a 5′ to 3′ orientation

Assay	Primer name	Primer sequence (5′ −> 3′)
LAMP	F3	AAATGCCAGGTTCGATCA
	B3	TCTTAGGTGAATTACCTGTACG
	FIP	TGCATTAGCTCAAAACTAATTGGGT-GAACTGCATATCCATATATTCTCC
	BIP	GCTATTCCTGAAACTTTTGAAACGT-CAAGTCTTGCCTTGAAGGA
	LF	TAGATCTTGCGAATTGATAGGGGT
	LB	GCAAAGAAAGAACGTTTGGTCATCT
RPA	TwstoMndo3 Fwd	CTACTATGGAGTGCTAGGGGCAGTTAATTGAG
	TwstoMndo5 Rev	TCCACTGAAAAATATCGTCTTAGGTGAATTACCTG
qPCR	TC Universal CS Fwd	TGTCCTACATATCTCAAGTCCCATTT
	TC Universal CS Rev	AAGGGTAGCTCCGGCTTCAA
	TC Universal CS TaqMan probe	/5HEX/TA+GTAGA+CTTGA+GAAA+CATGC/3BHQ_1/
	MADC2 Fwd	GTGACGTAGGTAGAGTTG
	MADC2 Rev	GTGACGTAGGCTATGAGAG
	MADC2 TaqMan probe	/56-FAM/CT+AAT+GCA+GG+CTATT+CC/3BHQ_1/
HRM	Choco Mando Fwd	CTGCATATCCATATATTCTC
	Choco Mando Rev	GTGACGTAGGCTATGAGAG
	Break 1 Fwd	TGGTCCGCAAAGAAAGAACGTTTG
	Plus 9 Rev	GATCAAACCTATTTATGGCATTTAC
	MADC2 Fwd	GTGACGTAGGTAGAGTTG
	MADC2 Rev	GTGACGTAGGCTATGAGAG
	−403 Fwd	GAGAAATTTCTTTGATTAGCAAAG
	−237 Rev	GGACTATTTATAGTCGCAAGGATG

Pre-amplification reactions were diluted 1:5 with 100 μL nuclease-free water (Ambion, Austin, TX, USA). Diluted pre-amplification, reactions were prepared for quantification using the QuantiFluor dsDNA System (Promega, Madison, WI, USA) and quantified using a Quantus Fluorometer (Promega, Madison, WI, USA) as per manufacturer’s instructions. Quantitated diluted pre-amplification reactions revealed final working concentration of ~1 ng/μL, which were used as non-normalized input into the real-time qPCR or HRM reactions.

### Quantitative real-time PCR TaqMan analysis

The qPCR analysis was performed in 10 μL reactions on a LightCycler 480 qPCR (Roche Applied Systems, Pleasanton, CA, USA) using the following protocol: (1) pre-incubation cycle (95 °C for 20 s), 45 amplification cycles (95 °C for 1 s, 60 °C for 20 s, 72 °C for 20 s) with a single acquisition mode setting for each cycle at 60 °C annealing, followed by a final cooling cycle (40 °C for 30 s) (Klimyuk et al. 1993).

Each reaction contained 5 μL of ~1 ng/μL of the diluted pre-amplified template used as input, 5 μL of TaqMan Master Mix (prepared per reaction as follows: 3.75 μL of FastTq Advanced Reaction Mix (Applied Biosciences, Pleasanton, CA, USA), 2.25 μL sex test primer mix (MADC2 fwd/rev at 3.33 μM and TC Universal CS fwd/rev at 0.167 μM working concentration), and 0.1875 μL of TC Universal CS TaqMan probe/MADC2 TaqMan probe (10 μM working concentration).

qPCR data was analyzed using the LightCycler 480 software AbsQuant/2nd Derivative Max algorithm for calculating Cp values.

Accumulation of fluorescent signal in target FAM and reference HEX wavelength channel results in cycle quotient values that increase in fluorescence signal as target DNA element copies are accumulated. Once the fluorescent signal passes an instrument derived threshold (calculated using the 2nd derivative max of the cycle threshold), the wavelength channel signal is called positive for the sample, and the cycle at which this occurs is measured and reported. The absence of fluorescent signal results in no measurement of a cycle quotient value. Detection of cycle quotient value for both MADC2 Taqman probe (FAM) and for TC Universal CS TaqMan probe (HEX) DNA elements complementary to the fluorescently tagged TaqMan assay probes results in ID for true genetic males, while the absence of MADC2 DNA element and the presence of cycle quotient value for cannabinoid synthase control probe result in identification of not male *Cannabis* individuals.

### High-resolution melt (HRM) analysis

HRM analysis was performed in 10 μL reactions on a LightCycler 480 qPCR (Roche Applied Systems, Santa Clara, CA, USA) using the following protocol: (1) pre-incubation cycle (95 °C for 10 min), 45 amplification cycles (95 °C for 10 s, 60 °C for 15 s, 72 °C for 10 s), 1 cycle of HRM (95 °C for 1 min, 40 °C for 1 min, 65 °C for 1 s, and heat to 95 °C with 25 continuous acquisitions per degree (C) followed by a final cooling cycle (40 °C for 10 s) (Klimyuk et al. 1993).

Each reaction contained 5 μL of ~1 ng/μL of the diluted pre-amplified template, 5 μL of HRM master mix (prepared per reaction as follows: 3.5 μL 2× HRM master mix containing HRM dye (Roche Applied Systems, Santa Clara, CA, USA), 0.6 μL of 4 μM primer mix, 0.8 μL of 25 mM MgCl2, 1.125 μL of nuclease-free water). Alternatively, if kit-purified DNA was used, 1 μL of purified DNA was used in replacement of the 5 μL of diluted pre-amplified NDA.

HRM data was analyzed using the LightCycler 480 melt genotyping software. Fluorescence intensity as a function of temperature for each sample also was analyzed using custom R scripts to determine statistical variation of melt curves and clustering of samples into the male and non-male genotypic classes.

### DNA amplicon sequencing

Male samples were sequenced using ThermoFisher’s SeqStudio capillary sequencer for MADC2 with the BigDye Direct (BDD) Cycle Sequencing kit (ThermoFisher, Fremont, CA, USA). M13 tailed end primers were designed to modify MADC2 Fwd and Rev primers with M13 Fwd and Rev oligonucleotides, respectively. Two reactions were prepared in a 96-well plate format for a forward and reverse read with 1 μL of 4 ng/μL genomic DNA used as template, 1.5 μL of 0.8 μM tailed end primer mix, 2.5 μL nuclease-free water, and 5 μL of BDD Master Mix to formulate a BDD PCR reaction using the BDD under the manufacturer’s instructions. The following PCR protocol was carried out on a Veriti Thermal Cycler (Applied Biosystems, Waltham, MA, USA): (1) hold hot start cycle (95 °C for 10 min), 35 amplification cycles (95 °C for 3 s, 60 °C for 15 s, 68 °C for 30 s), and a final extension (72 °C for 2 min) with an indefinite 4 °C hold. The BDD sequencing master mix was prepared with 2 μL of the BDD sequencing master mix and 1 μL of either one sequencing primer: BDD M13 Fwd primer or BDD M13 Rev primer.

### Loop-mediated isothermal amplification and isothermal recombinase polymerase amplification

LAMP and RPA sex determination assays were designed in house using Steep Hill male *Cannabis* genome assembly. In the RPA assay designs, biotin-labeled primers were designed for target amplification and detection. A standard reaction was prepared with reconstituting a TwistDX reaction pellet with 30 μL rehydration buffer, 2.1 μL Fwd and Rev primer each, 0.6 μL TwistMan target probe, 2.1 μL nuclease-free water, and 10 μL of crude DNA extract. A total of 2.5 μL 280 mM magnesium acetate is added to start the reaction (TwistDX, Cambridge, England). The reactions were incubated at room temperature for 30 min and then were applied to a disposable HybiDetect-2 strip and nucleic acid detection device type 3 (Milenia Genline, Germany). The results developed in 1–2 min following application. A positive male result is two bands detected, while a non-male-positive RPA reaction results in one detected band. In a similar workflow, LAMP primers were designed using Eiken’s LAMP PrimerExplorer V5 (Fujitsu Limited, primerexplorer.jp). Following optimization, a standard reaction was set up with 12.5 μL WarmStart LAMP 2× Master Mix, 2.5 μL LAMP primer mix 10× (16 μM FIP, 16 μM BIP, 2 μM F3, 2 μM B3, 4 μM LF, and 4 μM LB from Table 1), 1 μL DNA, and 8.5 μL nuclease-free water for a 25 μL final volume. Reactions were incubated at 65 °C for 30 min and measured for visual change in color indicating amplification of target male amplicon. Positive reactions accumulate amplicons and modify the pH which changed the color to yellow, while negative reactions do not and remain pink. New England Biolab standard LAMP PCR protocols were executed according to manufacturer’s instructions (NEB, Ipswich, MA, USA).

## Results and discussion

### Real-time qPCR sex determination assay in Cannabis sativa

**Fig. 1:**
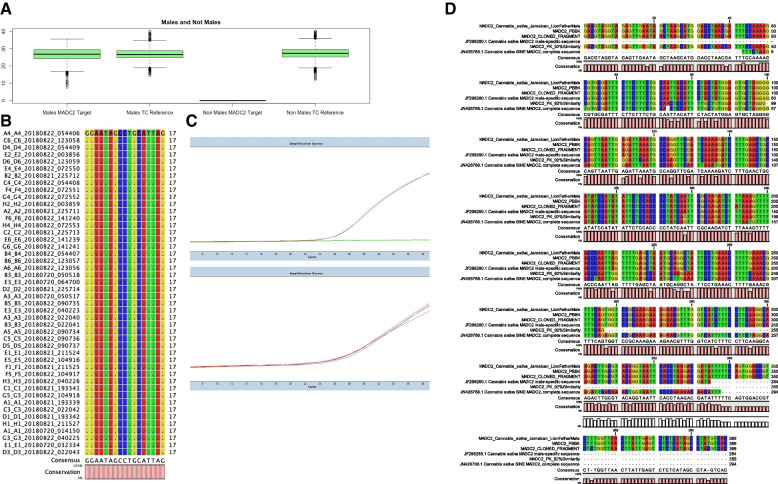
Male plant sex marker analysis by qPCR and sequencing. **A** Box plot analysis of 2nd derivative cycle quotient values for MADC2 and tissue control for 8200 samples from SexID qPCR results. **B** Genetic alignment of Steep Hill TaqMan probe for male identification from targeted sequencing of 45 males. **C** Representative amplification plots for Steep Hill sex determination assay (top: MADC2 amplification plot) (bottom: tissue control amplification plot). **D** Genetic alignment of canonical MADC2 391-bp cloned fragment from Mandolino aligned to queried IDs in PacBio sequence from Pineapple Banana Bubba Kush

A total of 8200 individual *Cannabis* plants have been genotyped using Steep Hill’s SexID assay with genetic material isolated either from an early leaf raw sample or an FTA card leaf imprint The SexID assay is a TaqMan real-time PCR in a duplex with custom-designed molecular fluorescent TaqMan probes: 5′FAM-3′BHQ labeled for MADC2 male detection and 5′HEX-3′BHQ for universal THCA/CBDA/CBCA synthase detection as a tissue control/*Cannabis*-specific reference gene controlling for detection of any *Cannabis* lineage irrespective of its cannabinoid synthase loci. An early validation set of 20 plants were germinated and characterized in house (Faux et al. 2014), analyzed by SexID qPCR assay, and conferred with observed phenotype (Supplemental Table 4). A box plot analysis of Cq (cycle quotient) values for *Cannabis* samples tested from 2015 to 2019 is reported Fig. 1A generated using the box plot function in the R ggplot2 package (Wickham 2009). Averages and population standard deviation of raw cycle quotient values were calculated (Supplemental Table 3) and reveal concordance between male and non-male cannabinoid synthase tissue control detection and MADC2 detection in males (average is thick black bar in Fig. 1A). The qPCR cycle quotient (Cq) value representative of MADC2 detection in 3156 males, the detection of tissue control cannabinoid synthesis for 8200 samples and the absence of MADC2 detection in 5047 non-males demonstrate a robust high-throughput real-time genotyping assay for *Cannabis*. We suspect that population skew towards female cannabis from our customer submissions influenced expected values, so we did not make comments on this dataset as it was a true breeding population. The 8200 samples coming to the lab were from various sources, breeding populations, and cultivation operations, not just traditional plant breeders. However, we have highlighted sex test genotype results (by HRM) in true breeding populations of European seeds tested at the CRAG from a given traditional cross, and we observed 40-45% male and 55-60% female as demonstrated in Supplemental Table 2. To confirm sequence specificity for the MADC2 molecular probe, targeted sequencing of MADC2 was performed using ThermoFisher SeqStudio capillary sequencing. Genetic alignment of targeted MADC2 amplicon sequence analysis reveals highly specific genomic homology and conservation at molecular probe loci for MADC2 from 45 randomly selected male samples matches canonical MADC2 isolated and cloned from male *Cannabis* by Mandolino and high homology to JF298280.1 *Cannabis sativa* MADC2 male-specific sequence submitted to GenBank ensuring accurate detection of genetically diverged male *Cannabis* individuals (Mandolino et al. 1999; Mandolino et al. 2002). Direct genomic sequence data available for the male sample Pineapple Banana Bubba Kush (BioProject no.: PRJNA378470) exhibits strain’s amplicon was otherwise identical to the consensus MADC2 sequence (Fig. 1B). Male MADC2 amplicon maps to 1 Mbp Y-chromosome contig in Jamaican lion male genome in addition to a relatively small 16-k bp fragment from the PBBK male genome (Supplemental Figs. 3 and 4) further indicate that the MADC2 male homology is a Y chromosome-associated fragment, and our target TaqMan probe detects Y-chromosome individuals carrying this genotype. The Y chromosome is considered homomorphic and degenerate with little to no homology or overlap with the X-chromosome and likely does not recombine (McKernan et al. 2020; Prentout et al. 2020a; Prentout et al. 2021).

### Assays as alternative sex ID methodologies

We developed sex determining specific designs around the Y-chromosome-specific region of MADC2 and designed several assays that can be used in the field to process and detect male DNA from early vegetative plants. We explored two different isothermal protocols including TwistDX primer and probe designs and LAMP PCR designs for each respective assay. In preliminary analysis using the TwistDx RPA system, we employed a custom designed TwistDX assay to analyze randomly selected samples of male and non-male *Cannabis* individuals from the 8200 sample test set. We designed custom twist primers and probe to target specific Y-chromosome DNA elements in a single step reaction at room temperature. In this assay, results are measured from test samples with two positive bands for the target amplicon, and assay control developed Milenia Genline HybriDetect-2 strip in a nucleic acid detection device type 3 for male samples and one assay control positive band (Milenia Genline, Germany). In our preliminary analysis of a small subset of *Cannabis* samples, we successfully identified male from non-male *Cannabis* individuals and water control (Supplemental Fig. 5B). We have also designed LAMP primers for MADC2 around the canonical sequence found in diverged males and carry out loop-mediated isothermal amplification using the WarmStart Colorimetric LAMP 2× Master Mix under manufacturer specifications. After 30 min at 65 °C incubation, male MADC2 DNA elements are copied, amplified, and accumulated if they are present; detection of target amplicon can be visually observed with yellow visual signal positively identifying genetic diverged male individuals and pink visual negative signals for non-male *Cannabis*. In a preliminary analysis of small sample size (*n* = 5 genetic individuals), tested in duplicate, we successfully identified males (yellow) from non-males (pink); two no template controls were used as negative input (Supplemental Fig. 5A). While this preliminary analysis demonstrates proof of concept for male detection using these two isothermal amplification technologies, additional testing and validation is needed though the assays show promise as viable commercial screening assays.

### High-resolution DNA melt analysis as a contextually effective proxy for sequencing and qPCR

**Fig. 2:**
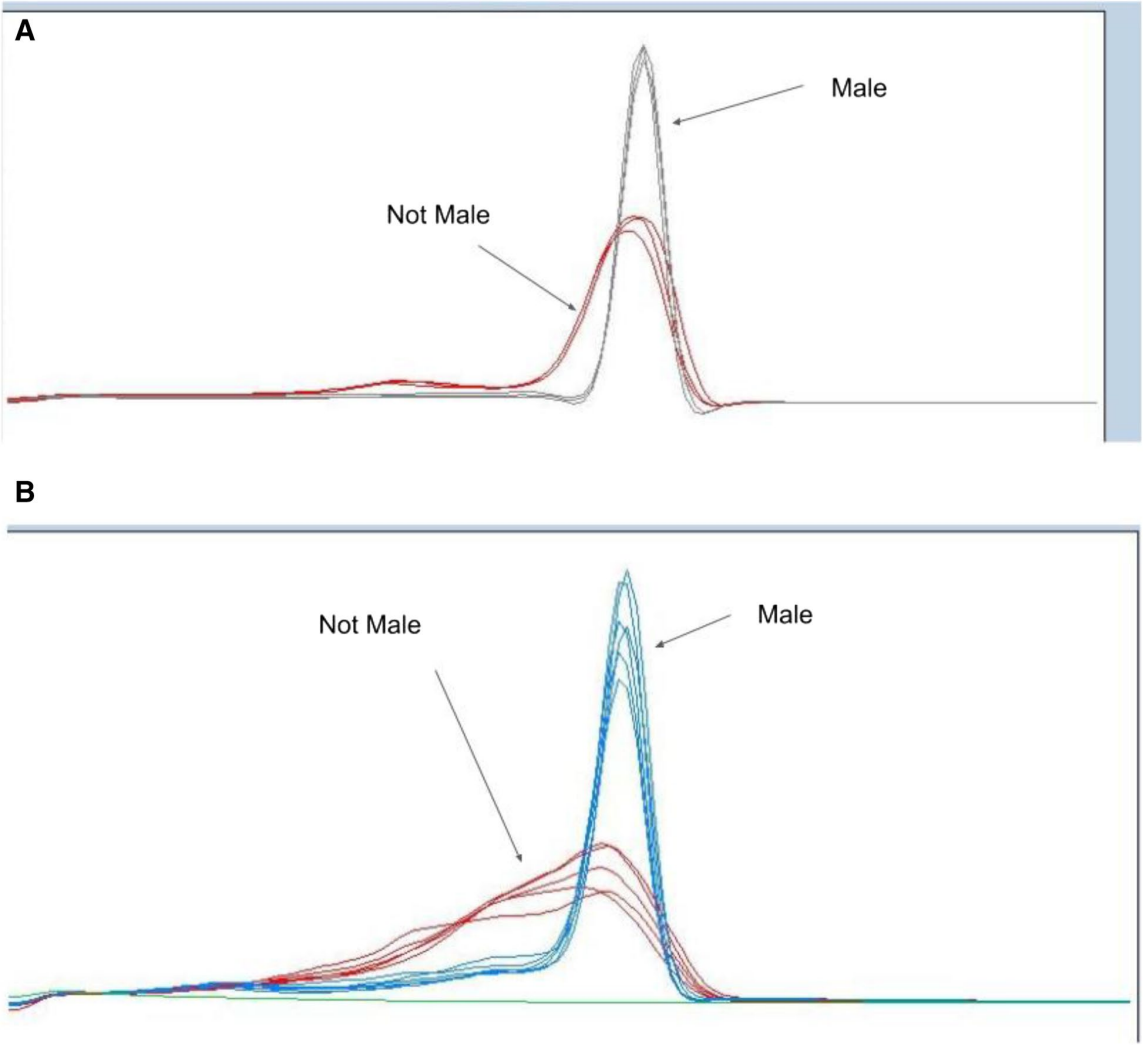
High-resolution melt (HRM) curve profiles from sex markers derivatized high-resolution melting analysis curves on selected *Cannabis sativa* male and not male samples from the SexID Steep Hill high-throughput testing sample set

HRM analysis provides a cost-effective and reproducible method to screen for novel sex-specific variants in a large population, particularly in non-male populations. By producing sex-specific amplicons, such as MADC2, from a variety of germplasm, HRM allows the user to understand genotypic differences through the temperature at which DNA transitions from double stranded (dsDNA) to single stranded (ssDNA)  (Vossen, van der Stoep and den Dunnen, 2007). By increasing the temperature in small increments over time, the relative fluorescence signal can be detected providing a definitive temperature at which the DNA melted apart. These temperature-specific fluorescence signals allow for the discovery of novel variants of males and non-males or if there are potential off-target amplicons from non-males that potentially provide a false result in a simple presence-absence reaction. Thus, these advantages suggest HRM as a superior method of screening for MADC2 and other sex-related elements, to provide a verification of the nucleotide signature encoding the male-specific element. Specifically, we have shown this differentiation of genotypes via HRM in both the literature-described MADC2 region (Mandolino et al. 1999; Mandolino et al. 2002) (Fig. 2A) and other non-previously described male-associated DNA (Fig. 2B) which shows that non-male genotypes are more likely to have variation in this targeted region, which may play a role in understanding sex determination in *Cannabis*, particularly with hermaphroditic and monoecious genotypes.

While HRM provides a more cost-effective and accurate result than previous methods, the melting temperature of these DNA amplicons is of particular importance in HRM analyses, especially in the considerations needed to interpret the data. The melting temperature (Tm) of any specific DNA sequence is conventionally defined as the temperature at which one of every two copies of that segment of DNA molecules is rendered completely single stranded by the thermal energy of the system (Lorenz 2012). While the specific Tm of a given DNA fragment is determined by its overall base composition, specifically the pyrimidine:purine ratio plays a particularly important role in addition to the various secondary structures of said amplicon, such as sites of potential formation of cruciforms, z-DNA, or other alternate secondary structures (Distefano et al. 2012; Blake 1987). These thermally relevant structural features can manifest as secondary peaks or shoulders in the melt profile for non-male individuals, sized in proportion to the fraction of the total sequence encompassed by the responsible irregularity and correspondingly detracting from, or slightly shifting the location of the main peak, while male individuals result in clearly defined and conserved HRM melting profile peak that is unshifted across different male cultivars. Therefore, HRM profiling for MADC2 was employed to rapidly and economically screen multiple genetic individuals to discriminate genetically conserved male amplicons from the various low homology versions MADC2 amplicons found in non-male cultivars. This process was also performed in a diverse set of 158 genetically unique individuals from European cultivars of varying lineages to ensure the robust and reproducibility of the assay being tested (Supplemental Table 2). Melt profiles of MADC2 were grouped by genotype and used to identify and associate MADC2 genotypes of males and not males. The plants were propogated and phenotyped (Faux et al. 2014) and example morphologies photographed for phenotypic reference (Supplemental Fig. 6). Nearby regions to the MADC2 locus, specifically the −403 Fwd and −237 Rev, were also investigated and presented a similar pattern of males having a high homology that differs from the more variable non-male genetic signature, showing that not only the MADC2 region is conserved but also nearby loci on the Y chromosome (Supplemental Fig. 2).

Figure 2 demonstrates a clear genotype difference observable in the second derivative melt curve analysis of two marker sets high-resolution melt profiles of male and non-male *Cannabis* plants: (A) high-resolution melt analysis for sex test (−403 Fwd + −237Rev) and (B) HRM sex test (MADC2 Fwd + MADC2 Rev). In Fig. 2A, the gray lines represent melt profiles of male genotypes, whereas the red lines represent melt profiles non-male genotypes. In Fig. 2B, the blue lines represent melt profiles of male genotypes, whereas the red lines represent the melt profiles of non-male genotypes.

## Conclusions

In this study, we investigated several methodologies for performing high-throughput sex identification genotyping in *Cannabis* plants using both novel and literature marker sets. We expanded on Mandolino’s report of MADC2 male specificity and have found our application of male-specific MADC2 sequence and other Y-chromosome-associated sequence for male detection assays to be suitable for high performance and application of *Cannabis* testing. Our method is cost effective comparatively, and the markers described in this study provide comparable end genotype result of male and non-male detection. Furthermore, we were able to place these markers on the recently assembled Y chromosome and show the conservation or lack therefore of within the amplicons produced by these widely used markers. The accurate identification of sex is essential to large-scale production and breeding of *Cannabis*; thus, the multiple methodologies presented here allow for accurate, quick, and cost-effective screening that will enable future development of germplasm and the industry. Our several real-time assays that can be performed by *Cannabis* testing laboratories perform diagnostic testing as well as versions of the assays that can be used in the field with minimal molecular biology equipment and expertise allowing for a wide range of users and throughput options.

## Supplementary Information

**Additional file 1: Supplemental Figure 1.** High resolution melting profiles on selected Cannabis sativa male and not male samples from the SexID Steep Hill high throughput testing sample set of MADC2 primer set showing multiple non-male melt profile genotypes. The samples that are shown in red represent male calls with a Tm melting peak at 82°C, whereas the other colors (purple, blue, maroon) represent non-male genotypes with variable target melting peak. **Supplemental Figure 2.** High resolution melting profiles of a region upstream of MADC2. This analysis was performed using primers designated, -403 Fwd and -237 Rev, which target an upstream region of MADC2 on the proposed Y-chromosome. Curves with an inflection point at 78°C are indicative of a male genotype, and the other curves present represent the various non-male genotypes of the homologous region. **Supplemental Figure 3.** Genomic Scaffold and Amplicon Alignment on JL_Father’s Y-Chromosome. The above alignment shows the location of where the MADC2 region lies on a 1Mbp region of the JL_Father’s Y-chromosome scaffold. The highlighted region near 523Mbp shows the MADC2 probe binding site and the black bar represents the amplicon region. **Supplemental Figure 4.** Alignment of 42 male amplicon sequences to the MADC2 genomic region in the JL_Father genome that shows a high conservation between male genotypes of diverse drug-type and fiber-type cultivars. The highlighted region represents the probe binding site that is also conserved between all the genotypes. 011623 contig from PBBK aligned to 1Mbp Y_000295F contig from JL father with TaqMan probe highlighted 2) MADC2 aligned to Y contig from Jamaican Lion Father with TaqMan probe highlighted examined. **Supplemental Figure 5.** A. Preliminary Analysis of Loop mediated Isothermal PCR for Male (yellow) and not Male (Pink) identification. B. Twist Dx Assay of Male and Not Male DNA samples. **Supplemental Figure 6.** Example phenotypes of representative cannabis plants from the 158 European cultivars germinated, propagated, and tested at the CRAG. A. Male *Cannabis* plant expressing a male sex phenotype of immature staminate bearing flowers. B. Selected female trial plants grown in greenhouse post sex testing. C. Non male *Cannabis* plant expressing an alternative female sex phenotype of maturing pistallate bearing flowers with partial staminate bearing. **Supplemental Table 1.** SexID qPCR Results for 8595 *Cannabis* Samples – This table shows the Cycle quoient values observed for the SexID assay using the MADC2 Fwd + MADC2 Rev + MADC2 Taqman Probe. The sample IDs are representative of the Genkit lots that the samples as they were received. **Supplemental Table 2.** Results of 158 European Cultivars tested by HRM using the MADC2 primers. 71 out of 158 or 45% of the population was measured with the melting peak at 82°C indicative of the male MADC2 genotype, and variable target melting peaks were observed for the remaining 186 test samples indicative of a not-male MADC2 genotype. **Supplemental Table 3.** Cycle Thresholds/Quotients for MADC2 and the TC Cannabinoid Synthase Positive Control qPCR Primers. The average Cq Value describes the cycle at which a positive detection of the target amplification occurred. The Population standard deviation describes the standard deviation observed in that Cq value when ran on 5047 non-male samples for the MADC2 target and 8200 samples for the positive control. On analyzing our qPCR results we see an avg. Ct value of 26 with a standard deviation of 3 for our tissue control and MADC2 qPCR assays suggesting they are reproducible and within our acceptable range of median Cq +/- 10 for a specific and reproducible qPCR assay. **Supplemental Table 4.** A Validation set of 20 samples (M1-20) with two template controls (M21-22) and a no template control was tested with the SexID qPCR assay as a part of early assay validation. The validation plants were grown from seed, and phenotyped (Faux et al. 2014). The observed sex phenotype results were recorded and compared against the results obtained by the SexID assay. Plants with a positve MADC2 signal corresponded with plants with an observed male phenotype (staminate bearing flowers).

## Data Availability

All data generated or analyzed during this study are included in this published article and its supplementary information files.

## Abbreviations

BDD: BigDye direct
Ct/Cq: Cycle threshold/quotient
DNA: Deoxyribonucleic acid
dsDNA: Double-stranded DNA
FTA: Flinders technology associates
Fwd: Forward primer sequence
HRM: High-resolution melting
JL: Jamaican lion
LAMP: Loop-mediated isothermal amplification
MADC: Male-associated DNA from *Cannabis sativa*
PBBK: Pineapple Banana Bubba Kush
PCR: Polymerase chain reaction
qPCR: Quantitative polymerase chain reaction
Rev: Reverse primer sequence
RPA: Recombinase polymerase amplification
ssDNA: Single-stranded DNA
Tm: Melting temperature
