# Supplementary material for: High-throughput methods to identify male Cannabis sativa using various genotyping methods
Source: J Cannabis Res. 2022 Nov 2;4:57. doi: 10.1186/s42238-022-00164-7 (PMC9628020; doi:10.1186/s42238-022-00164-7)
Supplement: Supplementary file 1 — Additional file 1: Supplemental Figure 1. High resolution melting profiles on selected Cannabis sativa male and not male samples from the SexID Steep Hill high throughput testing sample set of MADC2 primer set showing multiple non-male melt profile genotypes. The samples that are shown in red represent male calls with a Tm melting peak at 82°C, whereas the other colors (purple, blue, maroon) represent non-male genotypes with variable target melting peak. Supplemental Figure 2. High resolution melting profiles of a region upstream of MADC2. This analysis was performed using primers designated, -403 Fwd and -237 Rev, which target an upstream region of MADC2 on the proposed Y-chromosome. Curves with an inflection point at 78°C are indicative of a male genotype, and the other curves present represent the various non-male genotypes of the homologous region. Supplemental Figure 3. Genomic Scaffold and Amplicon Alignment on JL_Father’s Y-Chromosome. The above alignment shows the location of where the MADC2 region lies on a 1Mbp region of the JL_Father’s Y-chromosome scaffold. The highlighted region near 523Mbp shows the MADC2 probe binding site and the black bar represents the amplicon region. Supplemental Figure 4. Alignment of 42 male amplicon sequences to the MADC2 genomic region in the JL_Father genome that shows a high conservation between male genotypes of diverse drug-type and fiber-type cultivars. The highlighted region represents the probe binding site that is also conserved between all the genotypes. 011623 contig from PBBK aligned to 1Mbp Y_000295F contig from JL father with TaqMan probe highlighted 2) MADC2 aligned to Y contig from Jamaican Lion Father with TaqMan probe highlighted examined. Supplemental Figure 5. A. Preliminary Analysis of Loop mediated Isothermal PCR for Male (yellow) and not Male (Pink) identification. B. Twist Dx Assay of Male and Not Male DNA samples. Supplemental Figure 6. Example phenotypes of representative cannabis plants from the 158 [file 42238_2022_164_MOESM1_ESM.zip › Supplemental Materials.docx]

# Supplemental Materials:


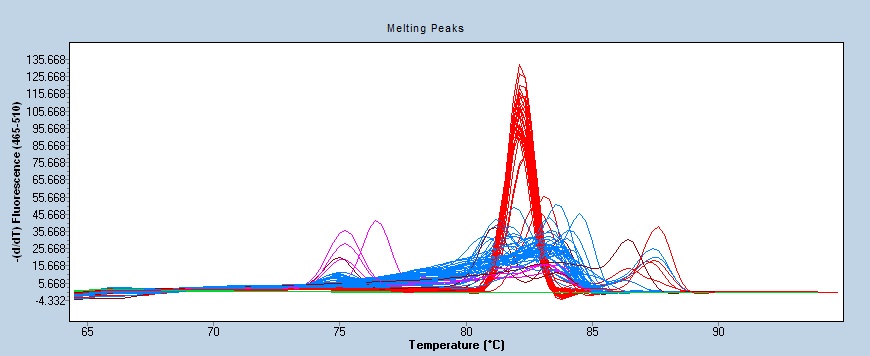


Supplemental Figure 1: High resolution melting profiles on selected Cannabis sativa male and not male samples from the SexID Steep Hill high throughput testing sample set of MADC2 primer set showing multiple non-male melt profile genotypes. The samples that are shown in red represent male calls with a Tm melting peak at 82˚C, whereas the other colors (purple, blue, maroon) represent non-male genotypes with variable target melting peak.

Supplemental Table 1: SexID qPCR Results for 8595 *Cannabis* Samples – This table shows the Cycle quoient values observed for the SexID assay using the MADC2 Fwd + MADC2 Rev + MADC2 Taqman Probe. The sample IDs are representative of the Genkit lots that the samples as they were received.


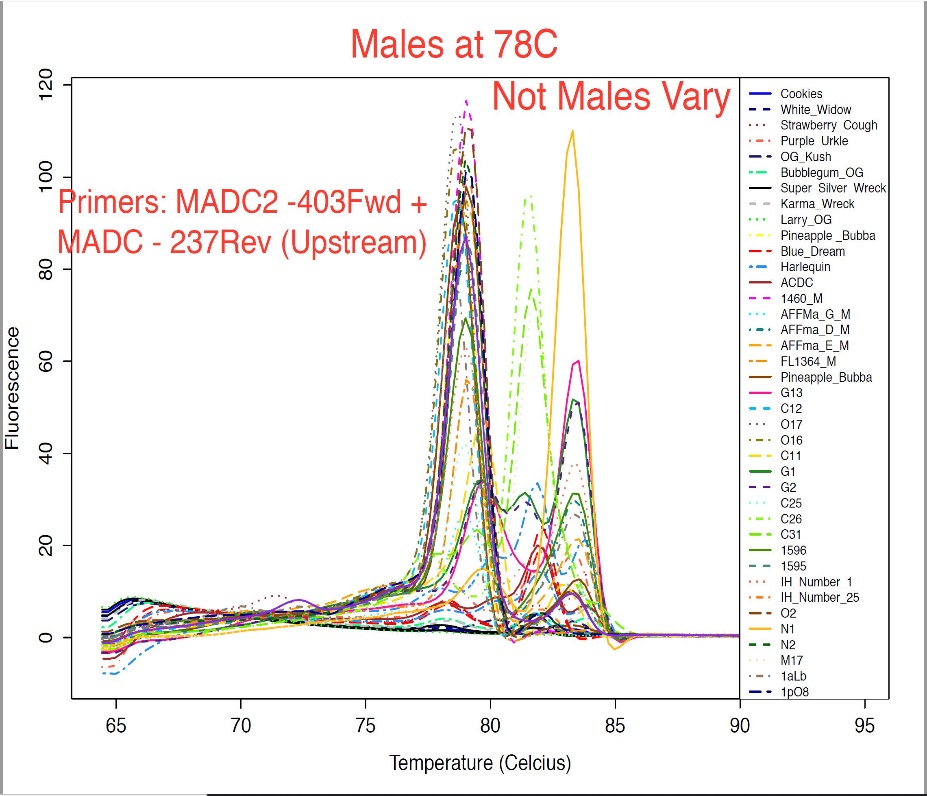


Supplemental Figure 2: High resolution melting profiles of a region upstream of MADC2. This analysis was performed using primers designated, -403 Fwd and -237 Rev, which target an upstream region of MADC2 on the proposed Y-chromosome. Curves with an inflection point at 78˚C are indicative of a male genotype, and the other curves present represent the various non-male genotypes of the homologous region.


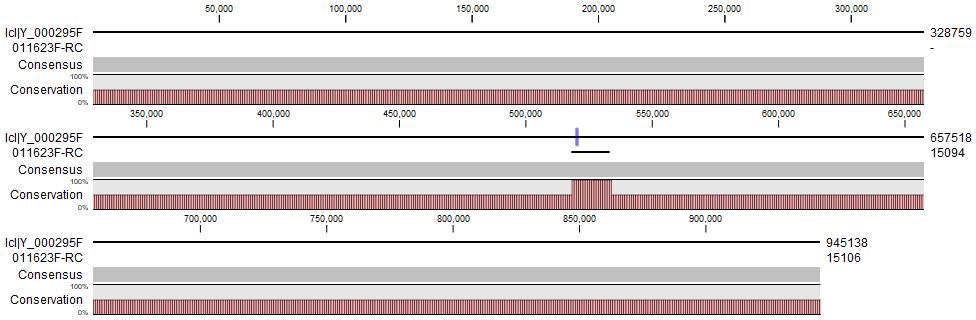
Supplemental Figure 3: Genomic Scaffold and Amplicon Alignment on JL_Father’s Y-Chromosome. The above alignment shows the location of where the MADC2 region lies on a 1Mbp region of the JL_Father’s Y-chromosome scaffold. The highlighted region near 523Mbp shows the MADC2 probe binding site and the black bar represents the amplicon region.
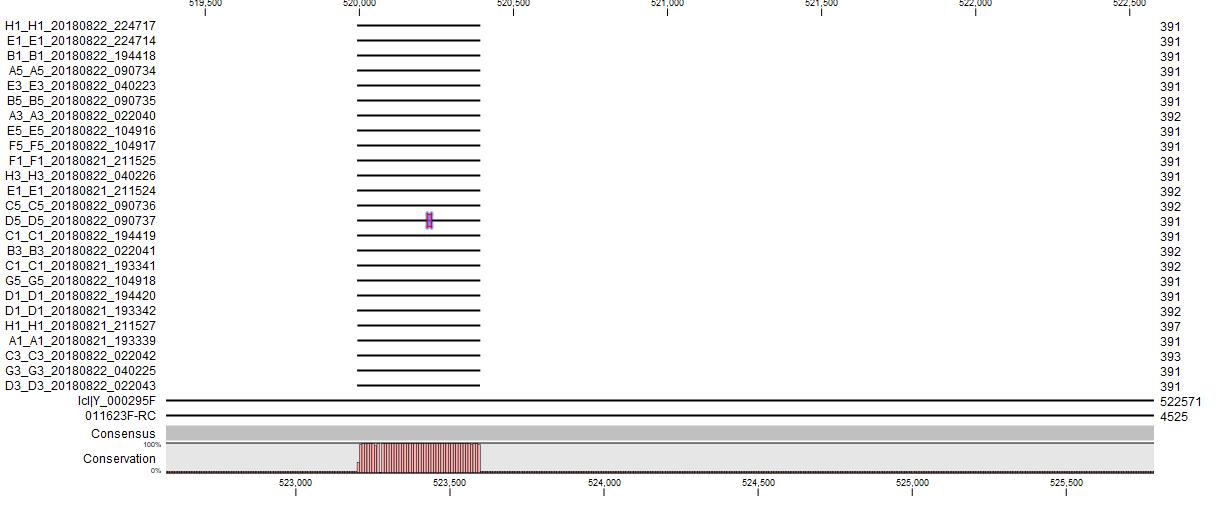
Supplemental Figure 4: Alignment of 42 male amplicon sequences to the MADC2 genomic region in the JL_Father genome that shows a high conservation between male genotypes of diverse drug-type and fiber-type cultivars. The highlighted region represents the probe binding site that is also conserved between all the genotypes. 011623 contig from PBBK aligned to 1Mbp Y_000295F contig from JL father with TaqMan probe highlighted 2) MADC2 aligned to Y contig from Jamaican Lion Father with TaqMan probe highlighted

examined.


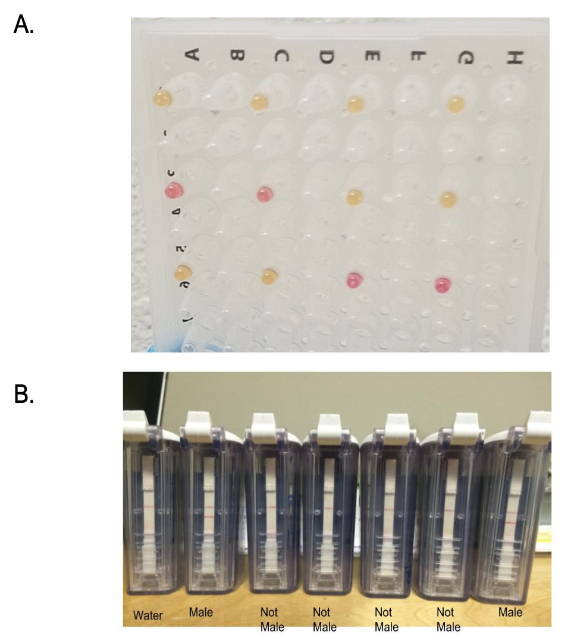

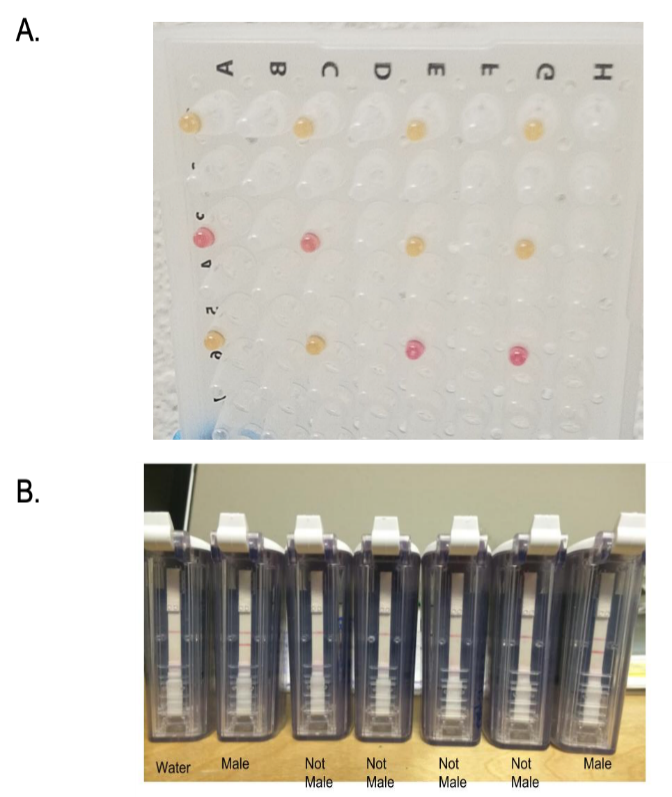


### Supplemental Figure 5:

Supplemental Fig. 5A. Preliminary Analysis of Loop mediated Isothermal PCR for Male (yellow) and not Male (Pink) identification. B. Twist Dx Assay of Male and Not Male DNA samples.

| HRM Group Color | \| 16744448 \| \| --- \| | \| 255 \| \| --- \| | \| 3200768 \| \| --- \| | \| 16711935 \| \| --- \| | \| 8421504 \| \| --- \| | \| 1234938 \| \| --- \| |
| --- | --- | --- | --- | --- | --- | --- | --- | --- | --- | --- | --- | --- |
| HRM Group Number | 1 | 2 | 3 | 4 | 5 | 6 |
| n | 71 | 4 | 78 | 2 | 1 | 1 |
| Sex | Male | Non-Male | Non-Male | Non-Male | Non-Male | Non-Male |

Supplemental Table 2: Results of 158 European Cultivars tested by HRM using the MADC2 primers. 71 out of 158 or 45% of the population was measured with the melting peak at 82˚C indicative of the male MADC2 genotype, and variable target melting peaks were observed for the remaining 186 test samples indicative of a not-male MADC2 genotype.

|  | MADC2  Target Cq | Cannabinoid Synthase  Reference Cq |
| --- | --- | --- |
| Average Cq Value | 24.222 | 26.979 |
| Population Std Dev | 3.233 | 3.170 |

### Supplemental Table 3:

Cycle Thresholds/Quotients for MADC2 and the TC Cannabinoid Synthase Positive Control qPCR Primers. The average Cq Value describes the cycle at which a positive detection of the target amplification occurred. The Population standard deviation describes the standard deviation observed in that Cq value when ran on 5047 non-male samples for the MADC2 target and 8200 samples for the positive control. On analyzing our qPCR results we see an avg. Ct value of 26 with a standard deviation of 3 for our tissue control and MADC2 qPCR assays suggesting they are reproducible and within our acceptable range of median Cq +/- 10 for a specific and reproducible qPCR assay.

| **Validation Sample ID** | **TC Universal Cannabinoid Gene Control qPCR Result (+\-)** | **MADC2 qPCR Result (+\-)** | **Observed Phenotype and Recorded Sex** |
| --- | --- | --- | --- |
| M1 | Positive | Negative | Female |
| M2 | Positive | Negative | Female |
| M3 | Positive | Negative | Female |
| M4 | Positive | Positive | Male |
| M5 | Positive | Negative | Female |
| M6 | Positive | Negative | Female |
| M7 | Positive | Positive | Male |
| M8 | Positive | Positive | Male |
| M9 | Positive | Negative | Female |
| M10 | Positive | Negative | Female |
| M11 | Positive | Negative | Female |
| M12 | Positive | Negative | Female |
| M13 | Positive | Negative | Female |
| M14 | Positive | Negative | Female |
| M15 | Positive | Negative | Female |
| M16 | Positive | Negative | Female |
| M17 | Positive | Negative | Female |
| M18 | Positive | Negative | Female |
| M19 | Positive | Negative | Female |
| M20 | Positive | Negative | Female |
| M21 | Positive | Negative | Female |
| M22 | Positive | Positive | Male |
| No Template Control | Negative | Negative | N/A |

Supplemental Table 4:

A Validation set of 20 samples (M1-20) with two template controls (M21-22) and a no template control was tested with the SexID qPCR assay as a part of early assay validation. The validation plants were grown from seed, and phenotyped (21). The observed sex phenotype results were recorded and compared against the results obtained by the SexID assay. Plants with a positve MADC2 signal corresponded with plants with an observed male phenotype (staminate bearing flowers).


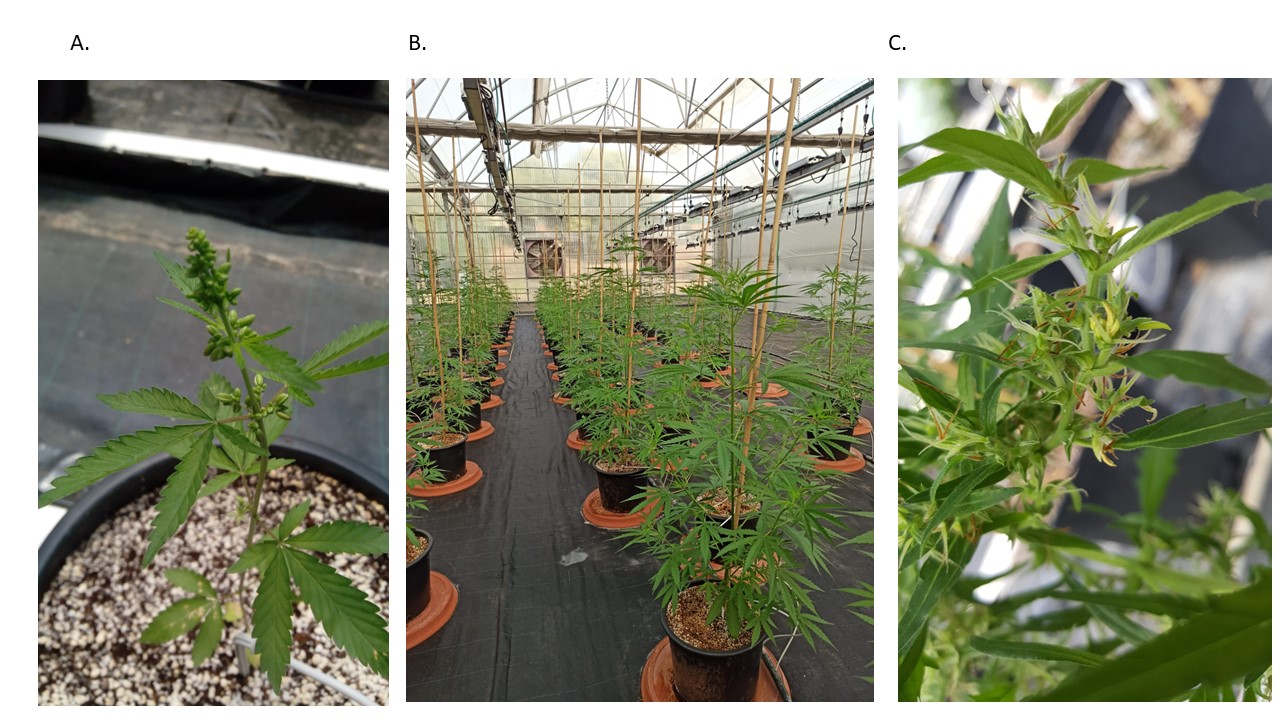


Supplemental Figure 6:

Example phenotypes of representative cannabis plants from the 158 European cultivars germinated, propagated, and tested at the CRAG. A. Male *Cannabis* plant expressing a male sex phenotype of immature staminate bearing flowers. B. Selected female trial plants grown in greenhouse post sex testing. C. Non male *Cannabis* plant expressing an alternative female sex phenotype of maturing pistallate bearing flowers with partial staminate bearing.
